# Conservative Laparotomy

**Published:** 1892-05

**Authors:** K. F. Slawiansky

**Affiliations:** St. Petersburg


DANIEL'S

### Texas Medical Journal.

A Representative Organ of the Medical Profession, and an Exponent of Rational Medicine; devoted to the Organization, Advancement and Elevation of the Profession in Texas.

Published Monthly.—Subscription $2.00 a Year.

Vol. VII. AUSTIN, MAY, 1892. No. 11.

### Original Articles.

CONTRIBUTED EXCLUSIVELY TO THIS JOURNAL.

*The Articles in this Department are accepted and published with the understading that we are not responsible for, nor do we indorse the views and opinions of the writers by so doing.*

Translated and Abbreviated from the Russian for Daniel's Texas Medical Journal.

### CONSERVATIVE LAPAROTOMY.

Being the President's address to the Gynecological Society of St. Petersburg, February 13, 1892.

BY PROF. K. F. SLAWIANSKY.

THE OBJECT of my paper is to direct your attention to the technical treatment of the inflamed pelvic organs of the female, by breaking up abnormal peritoneal adhesions, which operation may be termed *conservative laparotomy*.

The inauguration of this procedure marks a new era in the treatment of diseased ovaries and tubes. Time was, however, required for it to attain its present just pre-eminence. As all other important innovations, it had to undergo its period of trials and tribulations.

The removal of the uterine appendages for every kind of inflammatory disease seemed to be the rage, and the mellowing process of time was necessary to secure a reaction against such extreme measures. From a purely theoretical standpoint alone, a method which deprives a woman of her sexuality and mission in life, is not to be desired; for clearly it must not only be our task to alleviate the suffering of our patient, but, principally, to restore the functions of so important organs.

The radical operation to the extent that it has heretofore been performed, is therefore a lamentable eccentricity. The argument of the timid, that the breaking up of adhesions is a dangerous proceeding, will not hold good to-day. We know that it can be done with as much safety as any other intra-abdominal operation. It is, of course, necessary that the operator should be master of the technique. Only a total degeneration or destruction of these so eminently important organs justifies their total removal. It should consequently be our rule to preserve the healthy parts, and I consider it a fallacy to teach that healthy tissues should be sacrificed with diseased ones, for fear that they might themselves later become diseased. For this reason, I am opposed to the removal of the normal appendage in cases where the other one has had to be removed.

I contend that a woman with one sound ovary and tube is better off, under all circumstances, than one without any. In this opinion I am sustained by Landau. There is not sufficient evidence for the statement that a whole sound organ, or parts thereof, will deteriorate, if left behind. A. Martin reports twenty-one cases of resection of the ovaries where the sexual functions remained perfectly well preserved, menstruation remained normal, and in five cases pregnancy even took place.

Deserving of special attention, is the small cystic degeneration of the ovaries, which disease, in my opinion, is only a certain form of inflammation,—it is really an *oophoritis follicularis*, and does not call for a total removal of the organ. Recovery is possible, and takes place by atresia of the disturbed follicles, provided that no suppuration is present. Of course, where there is considerable purulent accumulation and threatening rupture, removal is necessary.

We ought to satisfy ourselves of the exact condition, and this should be done by an exploratory laparotomy, which enables us to make an ocular inspection of the organ, and to make a probatory and depleting incision through the whole length of the ovary, if deemed advisable, according to the teaching of Polk.

If there should be no new growth and no pus, then we may empty the follicles, if necessary, by puncturing them with the knife, and then close the ovarian incision with catgut. I have performed such an operation, with entire success, on a woman twenty-four years old, who suffered in the usual way, and on whom a prolapsed right and a considerably enlarged left ovary was made out. The operation consisted in a general breaking up of adhesions and a free incision through the left ovary, emptying the follicles, and then suturing the ovarian wound.

Prolapse of the ovary was also considered an indication for laparotomy. The suffering is here caused by adhesions of the organ within Douglas' pouch, which leads to torsion of the ligament, and consequently to passive blood stasis. Here, too, conservative surgery is indicated. Instead of being removed, the ovary should be freed.

Some ovarian malpositions are dependent upon those of the uterus, and therefore hysteropexia abdominalis may cure the former, of which fact I have had sufficient occasion to satisfy myself. But in cases where such a connection is not evident, often the mere freeing of the ovaries from their adhesions by breaking up the latter and by lifting up the ovaries out of Douglas' pouch is sufficient to cure both the distortion and the venous stasis. If, in such operations, the inclination of the ovaries, to fall back, be sufficiently evident to cause a fear of vicious reattachment, then the operation of Imlach and Polk ought to be added. The ovary is secured by carrying a catgut thread through its infundibular ligament, or through its hilus and then fastened to the pelvic peritoneum in a normal location.

The examples already enumerated show how in ovarian diseases the radical operation can be avoided. The same may be said of the fallopian tubes. Schroeder has already made attempts to reconstruct the permeability of atresic tubes, and though his efforts were ridiculed by Hegar, pregnancy has been made possible by resection of the atresic fimbrious ostium of the tube or by establishing a new ostium. In 1888, Martin operated in a case of oophoritis and salpingitis, by removing the right appendage, whilst he resected a portion of ahydrosalpinx on the left side, thus establishing here a new aperture. Menstruation became normal and in 1890 a three months fetus was aborted, whilst a re-examination, in 1891, showed a normal left appendage, both in size and sensitiveness. This case ought surely be incentive sufficient to stimulate us to further attempts in this direction. Let us hope that new methods will be found to open an atresic ostium and prevent its reclosing. Perhaps the latter indication may be filled by sewing a peritoneal fold around the brim of the fimbriæ. Another conservative method to cure salpingitis, where there is only slight ahesive inflammation, and in the absence of purulent accumulation, is the freeing, washing-out and cauterizating of the tubes, as practiced by Mundè and Polk.

To summarize then, there are many cases where castration can be avoided, though I am far from condemning it when necessary. There is no doubt either, that many a case of adhesive oophoritis and salpingitis can be cured by breaking up the abnormal adhesions; cases, where the destruction and degeneration of tissues has not yet taken place to such an extent as not to admit of restitution ad integrum. Here this mere act of freeing the organs and giving them their natural place and mobility, constitutes the operation. I will call it solutio adhæsionum perimetriticarum. It was first clearly defined and described by B. Hadra, of Texas, in 1885, and later also practiced with full premeditation and complete success by Polk, of New York. Horwitz, of Copenhagen, operated in 1889, and he, too, is of the opinion that in certain cases the healing act is not accomplished by removing the appendages but by freeing them from their surroundings. I have had the opportunity to satisfy myself of the correctness of this view in 1890. The result of the operation was so striking that Prof. Vulliet, of Geneva, who happened to be present, could not refrain from mentioning the case in his pamphlet: "Fifteen days in Petersburg."

The case is as follows: A woman, 26 years old, no children, complained of pain in her stomach and of daily vomiting for a whole year. An indistinct swelling, the size of a hen's egg, in the right parametrium. Operation—Excessive adhesions of all of the pelvic organs to each other and to the pelvic peritoneum. These adhesions were broken up. Nothing else was done. Immediately after the operation the vomiting ceased, pains subsided soon after and the patient regained perfect health.

The same operation restored the health of three other women, where the appendages in consequence of peritonitis were abnormally fixed.

From this brief epitome it may be seen that Hadra's operation is a typical representative of conservative laparotomy and that it is an operation which has already saved many a woman from being unsexed. His method has, as a matter of course, and as every new idea always does, found its opponents. Want ot understanding, and of good will to give it a practical trial, has caused some authorities to oppose and condemn the procedure. But inexorable time sets everything right.

During the six years following the publication of his article, the collective observation and experience have substantiated the correctness of Hadra's views, and his operation, which is beginning more and more to find its just recognition, has not only rescued many patient from suffering, but also from sexual mutilation.

